# Entrepreneurial intention, expectations of success and self-efficacy in undergraduate students of health sciences

**DOI:** 10.1186/s12909-022-03731-x

**Published:** 2022-09-15

**Authors:** Rita-Pilar Romero-Galisteo, Manuel González-Sánchez, Pablo Gálvez-Ruiz, Rocío Palomo-Carrión, Maria Jesus Casuso-Holgado, Elena Pinero-Pinto

**Affiliations:** 1 Department of Physiotherapy, Faculty of Health Sciences, University of Málaga, C/ Arquitecto Peñalosa, 29071 Málaga, Spain; 2 Department of Education, Faculty of Law and Social Sciences, International University of Valencia, Valencia, Spain; 3 Department of Physiotherapy, University of Castilla-La Mancha, Toledo, Spain; 4 Department of Physiotherapy, Faculty of Nursing, Physiotherapy and Podiatry, University of Sevilla, Seville, Spain

**Keywords:** Entrepreneurship, Entrepreneurial intention, Student, Higher education, Health sciences

## Abstract

**Background:**

Entrepreneurial intention is considered to be the best predictor of entrepreneurial behaviour. The Theory of Planned Behaviour (TPB) explains the degree of correlation between variables such as entrepreneurial intention, perceived feasibility and perceived desirability. Knowing the entrepreneurial intention of students of Health Sciences will help to guide and promote effective university policies to support entrepreneurship. The authors aimed to analyse the entrepreneurial intention of university students in the field of Health Sciences.

**Methods:**

A cross-sectional study was conducted in the Faculties of Health Sciences of two public universities of Southern Spain. 1518 students of different degrees of Health Sciences (Physiotherapy, Podiatry, Dentistry, Nursing and Occupational Therapy), from first to fourth year. An online structured questionnaire was used, the Entrepreneurial Event Model (EEM) adapted to the Spanish context. This measurement model was completed with 8 items from the Motivated Strategies for Learning Questionnaire-MSLQ.

**Results:**

The hypothetical model showed that perceived desirability and perceived feasibility were positive and significant predictors of entrepreneurial intention. Perceived desirability showed an indirect effect on entrepreneurial intention through perceived feasibility. Expectation of success and self-efficacy had no direct effect on entrepreneurial intention.

**Conclusions:**

Perceived desirability and perceived feasibility are related to entrepreneurial intention in Health Sciences students.

## Background

Entrepreneurship is a personal attitude that implies the need to grow through innovative actions and the capacity to recognise opportunities in the environment [1]. Therefore, being an entrepreneur involves both creating and diversifying, as well as intervening the already existing organisational processes [2]. Thus, it is considered a complex activity that combines cognitive, personal, social, economic, political and cultural factors, thereby constituting a multidisciplinary phenomenon. The analysis of the combination of all these factors helps to predict entrepreneurial intention [3]; in fact, it has been the most used model in the study of this phenomenon in the last years [4], since entrepreneurship is considered a fundamental element of modern economy due to its contribution to economic growth and employment creation [5].

Different models have been developed for the evaluation of entrepreneurial intention, with the Entrepreneurial Event Model [6] and the Theory of Planned Behaviour [7] being the most popular. In the former, the phenomenon of entrepreneurial event is influenced by desirability and feasibility, whereas, in the latter, entrepreneurship is influenced by the attitudes toward behaviour, the subjective rules and the perceived behaviour control. In any case, entrepreneurial intention is considered the best predictor of entrepreneurial behavior [8, 9],

In the context of Higher Education, entrepreneurship is defined from an integral human development approach, which allows generating employment from innovation and adaptation to the change demanded by society, favouring the relationship between business and the university [10]. However, although the creation of companies arouses interest in the scope of Higher Education [11], entrepreneurship as a scientific research programme is still under development [9, 12]. Therefore, enhancing entrepreneurship is a training complement in the university scope of many countries [13, 14, 15] and, although programmes that promote entrepreneurship have been fostered [16], it is fundamental to know the entrepreneurial intention of university students from the university itself [17]. Contemplating the development of specific competencies linked to entrepreneurship will increase the transfer of knowledge and the competitiveness of universities [18, 19].

Moreover, since Pintrich et al. [20] published the Motivated Strategies for Learning Questionnaire (MSLQ), motivational variables such as self-efficacy and expectation of success have been widely studied in different educational levels [21]. Expectation of success refers to the expectations of performance and is specifically related to task performance, whereas self-efficacy is an estimation of one’s capacity to carry out a task [21]. Although some researchers have studied the impact of self-efficacy on innovative behaviour in undergraduate students, this relationship remains unclear [22, 23]. In the specific literature on self-efficacy, expectation of success and entrepreneurial intention in the university context, few studies have delved into these aspects [24, 25].

In addition to the above mentioned, the health sector is facing an economic challenge. In the future, healthcare workers will need to adapt to the constant change and develop quality skills and solutions under economic restrictions [26]. A fundamental part of the free exercise of the profession is entrepreneurship, which is a competence derived from the functions of Health Sciences professionals [27, 28] and one of the fields studied in the GUESS report [29].

Knowing the entrepreneurial intention of the students of Health Sciences will thus help to guide and promote effective university policies to support entrepreneurship, in order to respond to the concerns and needs of both students and the job market. Likewise, such policies will help to develop specific competencies related entrepreneurship in the university. To the best of our knowledge, the relationship between these variables has not been jointly studied in the field of Health Sciences.

The aim of this study was to analyse entrepreneurial intention in university students of Health Sciences. To this end, we explored the path relationships between perceived desirability and perceived feasibility (Entrepreneurial Event Model), and between self-efficacy and expectation of success (Motivated Strategies for Learning Questionnaire), in order to determine their influence on entrepreneurial intention.

## Methods

A cross-sectional study was designed and developed in the Faculties of Health Sciences of two public Spanish universities. An online structured questionnaire was employed for data collection, and a hypothetical model related to entrepreneurial intention in students of different degrees of Health Sciences was created and tested.

### Participants

The participants were students of different degrees of Health Sciences (Physiotherapy, Podiatry, Dentistry, Nursing and Occupational Therapy), from first to fourth year. The inclusion criteria were as follows: being registered in any of the degrees of the Faculties of Health Sciences of the two participating universities, and having no difficulty at understanding and communicating in Spanish. The study excluded those who left one or more items unanswered. The composition of the sample resulted from a stratified sampling procedure, guaranteeing the representativeness of the students according to the classical differentiation by academic areas.

### Ethical considerations and procedure

This study was approved by the Ethics Committee of the University of Málaga (CEUMA 48–2019-H). Before providing the link to access the online questionnaire (lime survey by UMA-server), the participants were informed of the research and signed the informed consent form. In compliance with the Declaration of Helsinki, those who agreed to collaborate in the study were informed of the research objectives, guaranteeing their voluntary and anonymous participation by using study codes on the complete questionnaires, as well as the confidentiality of their answers. The researchers remained available in case any questions arose during the survey. The participants completed the questionnaire in 8–10 min. All methods were performed in accordance with the relevant guidelines and regulations.

### Instruments

We used the Entrepreneurial Event Model (EEM) [6], adapted to the Spanish context in a sample of former university students by Jaén and Liñán [30]. This instrument was then used in a sample of university students of the field of Education [22], obtaining adequate reliability and validity results. The EEM consists of three dimensions: perceived feasibility (6 items), perceived desirability (12 items), and entrepreneurial intention (5 items). Based on the procedure of Azjen [23], perceived desirability consists of two sets of 6 items each, which measure the expected results of a business degree and the convenience of such results. Therefore, a single dimension (perceived desirability) was created for the realisation of the analyses, consisting of 6 items that resulted from multiplying the expectations of results by their convenience and dividing the result by six to obtain the average scores of the dimension. All the items were evaluated with a 7-point Likert scale, ranging from low intention (0) to high intention (6).

The measurement model was completed with 8 items of the Motivated Strategies for Learning Questionnaire-MSLQ [24], which measure the expectation of success (4 items) and self-efficacy (4 items). The answers are gathered in a 7-point Likert scale (1 to 7), although we followed the structure of the EEM to avoid the confusion of the participants, obtaining a 7-point scale from “totally disagree” (0) to “totally agree” (6).

### Data collection and statistical analysis

Descriptive analyses were used to report the sociodemographic characteristics of the participants. Additionally, the normality of the data was calculated using univariate skewness and kurtosis values. We adopted the classic two-step evaluation [31, 32]: confirmatory factor analysis to test the psychometric properties for the measurement model and structural equation model to analyse the predicted hypothesised relationships between the variables of the present study. The internal reliability was tested by Cronbach’s alpha (α; [33]) and the internal consistency of the constructs was measured through composite reliability (CR [34];). According to Hu and Bentler[35], multiple goodness-of-fit indices were used to evaluate how well the proposed model in CFA and SEM fitted the data: Chi-square by degrees of freedom (χ^2^/df < 5; [36], Comparative Fit Index (CFI), Incremental Fit Index (IFI) and Tucker-Lewis Index (TLI) > 0.90 [35], the Parsimony Comparative of Fit Index (PCFI > 0.80; [37], and Root Mean Error of Approximation (RMSEA) < 0.08 [38, 39]. We assessed all analyses with SPSS and AMOS 21.0 package.

## Results

### Participant characteristics

The sample was composed of a total of 1544 students, of whom 26 were excluded for leaving a question unanswered (1.69%). Of the remaining 1518 participants, 1084 (71.4%) were female and 434 (28.6%) were male. In terms of age, 1131 (74.5%) were between 17 and 21 years old, 289 (19.0%) between 22 and 26 years, 44 (2.9%) between 27 and 31 years, and 54 (3.6%) were aged over 31 years. With regard to the academic degree in progress, 554 (36.5%) were nursing students, 383 (25.2%) studied physiotherapy, 260 (17.1%) studied podiatry, 174 (11.5%) were students in dentistry, 136 (9.0%) were occupational therapy students, and 11 (0.7%) were studying other degrees. Of the total sample of participants, 312 (20.6%) were both studying and working.

### Descriptive statistics and internal consistency reliability

The values for univariate skewness and kurtosis were satisfactory within the conventional criteria for normality: -3 to 3 for skewness and -7 to 7 for kurtosis [40]. The internal consistency for the different dimensions was adequate and higher than 0.7, specifically between 0.80 and 0.93 for Cronbach’s alpha and between 0.81 and 0.95 for composite reliability. With respect to the validity test, the average variance extracted for all dimensions exceeded the generally accepted value of 0.50 [34], with values between 0.56 and 0.84.

### Confirmatory factor analysis

The findings of the fit of the index using maximum likelihood estimation were adequate: χ^2^/df = 6.99; CFI = 0.94; IFI = 0.93; TLI = 0.93; PCFI = 0.83; RMSEA = 0.063. It is necessary to indicate that the χ^2^ statistic has been previously shown to be sensitive to the sample size [41]. However, the analysis of the factor loadings suggested the deletion of one item of the entrepreneurial intention scale (EI_3: 0.41). The remaining factor loadings exceeded the established criteria of 0.50 [34] and were statistically significant. In addition, on the basis of the analysis of the modification indices (MI) and theoretical knowledge, covariances between pairs of errors were included: perceived feasibility 2 and 4 (MI = 129.44) and entrepreneurship intention 1 and 5 (MI = 48.31). As a result, better fits were generated: χ^2^/df = 5.69; CFI = 0.96; IFI = 0.95; TLI = 0.95; PCFI = 0.83; RMSEA = 0.056. Table 1 shows the results of the composite reliability, Cronbach’s alpha, and correlations among the five variables, where results below 0.85 (ranging from 0.16 to 0.60) indicate evidence of the discriminant validity of the measure.

**Table 1:** Correlations and internal consistency of the latent variables

	CR	α	1	2	3	4	5
1.Perceived Desirability	0.81	0.80	0.52				
2.Perceived Feasibility	0.93	0.93	0.48*	0.69			
3.Self-Efficacy	0.83	0.85	0.32*	0.23*	0.65		
4.Expectation of success	0.88	0.87	0.31*	0.24*	0.52*	0.56	
5.Entrepreneurial intention	0.95	0.95	0.46*	0.60*	0.17*	0.16*	0.84

^*^ *p* < 0.01; average variance extracted in the diagonal

### Structural equation model and path analysis

**Fig. 1:**
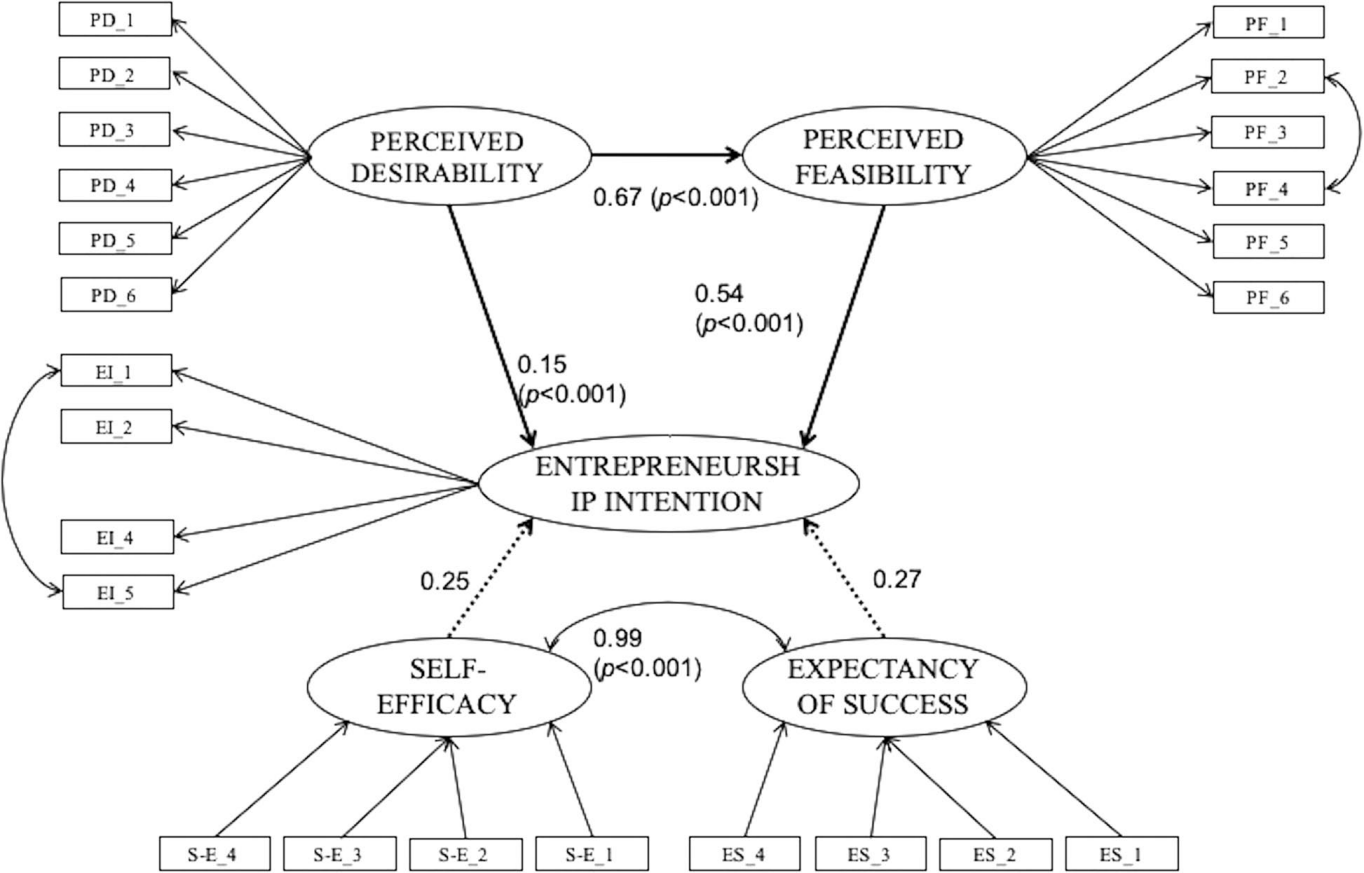
Path relationships of the final model (dotted lines: *p* > 0.05)

The SEM involved the relationships between the different dimensions of the measurement model, and the findings showed a good level of fit: χ^2^/df = 6.94; CFI = 0.94; IFI = 0.94; TLI = 0.93; PCFI = 0.82; RMSEA = 0.063. Figure 1 presents the structural relationships with the standardised estimates among the path relationships. The hypothetical model established that perceived desirability (β = 0.15; *p* < 0.001) and perceived feasibility (β = 0.54; *p* < 0.001) were positive and significant predictors of the entrepreneurial intention. Furthermore, perceived desirability showed an indirect effect on entrepreneurial intention through perceived feasibility (β = 0.67; *p* < 0.001). Expectation of success and self-efficacy had no direct effect on entrepreneurial intention: β = 0.27 (*p* < 0.49) and β = 0.25 (*p* < 0.52), respectively.

## Discussion

This study analyses the relationship between perceived desirability, perceived feasibility, entrepreneurial intention, self-efficacy and expectation of success. Based on the survey data provided by the large study sample, the results showed that perceived desirability and perceived feasibility are associated with entrepreneurial intention in Health Sciences students. This study also reveals that self-efficacy does not have a significant impact on entrepreneurial intention. Contrarily, Shen et al. [18], in their study conducted with nursing students, showed that self-efficacy could act as a mediator in innovative and entrepreneurial behaviour. These results differ from those reported in a previous study [31], where, in a sample of 304 university students of Business Administration, Commercial Engineering, Political Sciences, Law, Graphic Design, Education, Engineering and Psychology in Bolivia, they analysed the participants’ entrepreneurial intention, aptitude for change (innovation), level of ego-resiliency and self-efficacy. Of the total sample, 54.6% obtained high scores in entrepreneurial intention and a statistically significant correlation with self-efficacy, aptitude for change and ego-resiliency. Despite the fact that these results differ from those obtained in our study, it is worth highlighting that the academic profiles of the participants are very different between the two studies.

Regarding the analysis of perceived desirability and feasibility, both the study of Grimaldi-Puyana et al. [22] and that of Lara-Bocanegra et al. [8] reported a positive relationship between desire and viability, desire and entrepreneurial intention and viability and entrepreneurial intention, with perceived desirability and perceived feasibility being significant predictors of entrepreneurial intention. These conclusions came from a sample of university Sport Science students of a region of Southern Spain. Although the main conclusions drawn in the present study are in agreement with those of the aforementioned studies, the academic profile of the participants differs in the study population. Despite the fact that it was not an objective of this study, it is interesting to highlight the influence of sociodemographic and cultural variables on entrepreneurial intention [30]. However, very little attention has been paid to the cultural and social environment as a determinant of the characteristics of the workforce and their entrepreneurial attitudes [3, 13, 15]. The study carried out by Wardana et al. [42] reveals that the entrepreneurial attitude plays an essential role in mediating both entrepreneurial education and self-efficacy toward the entrepreneurial mentality of students. The findings of the present study also indicate that entrepreneurship education successfully influences entrepreneurial self-efficacy, entrepreneurial attitude, and entrepreneurial mindset. Therefore, it becomes clear that a well-incorporated entrepreneurial education is necessary at the university, also in health sciences students. For example, in Spain, only 4.5% of university students plan to become entrepreneurs when they finish their studies [29].

Regardless of the sociodemographic variables, Kostoglou and Siakas [12] analysed the characteristics of entrepreneurs with university degrees in Greece. Through a qualitative study, they analysed the factors that favoured entrepreneurship. They found that, among the degrees of Health Sciences, entrepreneurship was more relevant in physiotherapists (26.2%), followed, with a great difference, by midwives (2%) and nurses (1.6%). Although they analysed variables such as sex, specialisation, faculty or area and postgraduate studies, their results showed that the Faculty of Health Sciences obtained the lowest proportion of entrepreneurs (9.4%).

Among the participants of the present study, 20.6% were working and studying, thus these findings have important implications for educational settings, as they suggest that the reinforcement of learning with interdisciplinary teaching should be integrated into the curriculum, which could facilitate student entrepreneurial intention, according to Liu et al. [38].

For maximum benefit to students, strategies to address networking, inter-disciplinarity, and entrepreneurship must be embedded in the culture that research students experience throughout their research degree programme [43]. Moreover, since the economic crisis of 2008, the field of social entrepreneurship has experienced a considerable boom [44]. The study of Chien-Chi et al. [45], who analyzed the relationship between college students' emotional competences, entrepreneurial self-efficacy, and entrepreneurial intention, showed that socio-emotional competence has a positive effect on entrepreneurial intention; moreover, all dimensions of entrepreneurial self-efficacy were significantly and positively correlated with entrepreneurial intention. Therefore, it would be convenient to reinforce these ideas from the university [29].

The present study shows the reality of two different universities with some common degrees (Nursing, Physiotherapy and Podiatry) and other degrees which are taught in one of them (OT and Dentistry), all of which belong to the area of Health Sciences. Furthermore, the use of SEM (Structural Equation Modelling) to analyse and measure the relationship can provide a perspective that is increasingly popular in Health Sciences, with the subsequent advance of research in these fields of knowledge [39].

One of the main strengths of the present study is the large sample of participants, as well as the very small number of missing data, which means that the extracted data are solid. On the other hand, although the sample is homogeneous regarding the analysed degrees and the place of origin of the participants, it would be interesting to analyse other population groups, in order to identify consistencies and differences, which would help to design intervention plans that could potentiate entrepreneurship among university students. In any case, this investigation is relevant, as it introduces SEM in a specific study in Health Sciences, and it also supports the conclusions of previous studies.

Future studies could add qualitative research and analyse whether entrepreneurial intention results in the creation of business. Similarly, it would be interesting to study whether the changes in the possible curricula and university policies could pay off by increasing the number of companies created by students of Health Sciences. Likewise, exploring entrepreneurial intention from the gender perspective would provide interesting conclusions to our research field. Future research should involve public and private universities in Spain, in order to increase the diversity and generalisability of the research results.

Practical and theoretical contributions.

Some studies [26] reveal that the attitudes of university students toward entrepreneurship education have a positive and significant effect on their entrepreneurship self-efficacy. Participating in entrepreneurship courses has both an individual and a peer effect [46]. Thus, we suggest that entrepreneurship education in colleges should focus on students' attitudes toward it. Studies such as the present work, which delve into the aspects that influence entrepreneurship, should be taken into account when designing and formulating university strategies to promote entrepreneurship.

Furthermore, we also suggest that there should be specific entrepreneurship education for health sciences students at universities and that individual differences between students and different university degrees (specific disciplines) should be taken into account. In addition, gender differences and differences in the resource base of students' families must also be taken into account. In relation to this, the study carried out by İspir et al. [11], focused on the association of personality traits and entrepreneurial tendencies with the professional adaptability of nursing students, reveals that there is a positive correlation between the entrepreneurial tendency and professional adaptability. Students with high entrepreneurial tendencies have better career adaptability. They suggest that education should improve entrepreneurial traits, considering the personality traits of students, to ensure their adaptation to the health science profession.

## Conclusions

We found that perceived desirability and perceived feasibility are associated with entrepreneurial intention in Health Sciences students. These findings could help to design university policies that stimulate commercial activity among students, thereby contributing to the development of modern knowledge economy. Similarly, our results will help to develop specific competencies related to entrepreneurship in the university. Entrepreneurship can increase the visibility of the health profession and encourage the creation of new spaces for action for future health science professionals.

All these findings suggest that, first of all, the university needs to incorporate entrepreneurship into the curriculum of health sciences students, bringing in entrepreneurial health professionals as instructors. In addition, as is expected, it will be possible to modify and influence the attitudes of students with entrepreneurial intention from the university.

Finally, the university must support students in the formation of an entrepreneurial mindset.

## Data Availability

The datasets used and/or analysed during the current study are available from the corresponding author on reasonable request.
